# Evaluation of Asthma Course in Patients Hospitalized in Pediatric Intensive Care Unit Due to Severe Asthma Exacerbation

**DOI:** 10.3390/medicina61020341

**Published:** 2025-02-14

**Authors:** Ahmet Selmanoglu, Hatice Irmak Celik, Cankat Genis, Esra Kockuzu, Zeynep Sengul Emeksiz, Emine Dibek Misirlioglu

**Affiliations:** 1 Department of Pediatric Allergy and Immunology, University of Health Sciences Ankara City Hospital, 06800 Ankara, Turkey; dr.irmakcelik@gmail.com (H.I.C.); cankatgenis@gmail.com (C.G.); drzeynep83@hotmail.com (Z.S.E.); edibekm@yahoo.com (E.D.M.); 2 Department of Pediatric Intensive Care Unit, University of Health Sciences Ankara City Hospital, 06800 Ankara, Turkey; esrakockz@gmail.com

**Keywords:** asthma, PICU, severe asthma exacerbation, pediatric asthma, asthma management

## Abstract

*Background and Objectives*: Childhood asthma represents a significant global public health issue and is the most common chronic disease among children. Hospitalization costs, especially for intensive care, are quite high. This study aimed to evaluate the characteristics, prognosis, and preventable risk factors of patients admitted to the Pediatric Intensive Care Unit (PICU) due to severe asthma exacerbations. *Materials and Methods*: We assessed patients admitted to the Ankara Bilkent City Hospital PICU from January 2013 to December 2022 diagnosed with asthma based on The Global Initiative for Asthma (GINA) criteria. The collected data encompassed demographic and clinical characteristics, intensive care treatments, hospitalization duration, atopic conditions, and respiratory viral panel results. The current clinical status was assessed using hospital records and caregiver interviews, with a focus on recent emergency admissions, ongoing treatments, exacerbation frequency, and asthma control based on GINA guidelines. *Results*: The study comprised 83 patients with a mean age of 72.9 (±45.5) months, predominantly male (63.9%). The average follow-up duration post-discharge was 40.7 ± 26.9 months. Patients received respiratory support in the PICU for a mean of 3.8 (±2.8) days and systemic steroid therapy for 4 (±1.5) days. Respiratory viral panel results identified pathogens in 42 patients, with rhinovirus being the most frequent. Post-discharge, 72.3% of patients continued follow-up at pediatric allergy clinics. Of the 60 patients contacted, 67.5% were on current asthma treatment and 48.2% had experienced an exacerbation in the past year. Asthma management steps remained unchanged for 33 patients, decreased for 13, and increased for 47 (44.6%). Asthma maintenance treatments pre-admission and post-discharge showed that 44.6% (*n* = 47) of the patients required an increase in their GINA treatment step after PICU admission, which was statistically significant (*p* < 0.001). History of atopic dermatitis was a significant risk factor for escalating treatment steps in both univariate and multivariate analyses (*p* = 0.018, *p* = 0.03). *Conclusions*: We found that admission to the PICU due to severe asthma exacerbation not only increases the risk of recurrent asthma exacerbations but also serves as a risk factor for stepping up maintenance treatment according to GINA guidelines during long-term follow-up.

## 1. Introduction

Asthma is a heterogeneous chronic inflammatory disease characterized by reversible airflow obstruction and airway hyperresponsiveness. This condition can be triggered by a variety of factors, including allergens, infections, and pollutants [1,2]. Asthma management involves a range of therapies, including biologics, particularly in cases of severe asthma [3]. Childhood asthma is a serious public health problem around the world [4]. Asthma not only impacts a child’s quality of life but also poses significant economic burdens due to healthcare costs and missed school days for both children and their caregivers [5]. Among children, asthma is the most common chronic disease, ranking among the top 20 conditions worldwide for disability-adjusted life years in this age group [6]. Severe acute asthma is defined as a critical exacerbation of asthma that fails to respond adequately to standard treatments, including bronchodilators and systemic corticosteroids. This condition carries a significant risk of progressing to respiratory failure and, in some cases, may result in fatal outcomes [7,8]. Admissions to Pediatric Intensive Care Units (PICUs) for asthma exacerbations, while relatively rare, are significant indicators of severe asthma and inadequate management. Factors increasing the risk of PICU admission include a history of previous PICU stays, the need for mechanical ventilation, rapidly worsening exacerbations, uncontrolled asthma, and poor adherence to controller medications. The need for PICU care in a child with asthma indicates that standard management strategies have failed to control the exacerbation and highlights the severity of the condition [8,9,10].

The identification of key triggers that increase the risk of exacerbations in patients should be a primary objective for clinicians [11]. Multiple studies have investigated the risk factors for PICU admissions. Key determinants identified include adherence to regular and correct use of inhaled corticosteroids, increased frequency of emergency department visits, greater reliance on rescue medications, sensitization to aeroallergens, and exposure to environmental tobacco smoke. [12,13]. Asthma management includes the consistent use of controller medications and the use of bronchodilators for immediate symptom relief. Adherence to the prescribed treatment regimen is critical for optimizing therapeutic outcomes. Non-adherence has been associated with adverse effects, including the exacerbation of asthma symptoms, increased healthcare costs, compromised quality of life, and even mortality. In pediatric populations, adherence rates are often below 50%, contributing to significant asthma-related complications, including a higher incidence of intensive care unit admissions [14,15]. This underscores the importance of comprehensive asthma management strategies, including preventive measures, appropriate medication use, regular follow-ups, and patient education, to minimize the risk of severe asthma exacerbations and hospitalizations [16]. In the literature, we know that experiencing a severe asthma attack is a risk factor for having another severe attack in the following period. However, studies providing insights into the course of asthma following a severe asthma exacerbation requiring intensive care unit admission are limited in the literature. Our study was designed to examine the long-term effects of severe asthma exacerbations requiring PICU admission on the course and management of asthma in the pediatric age group.

## 2. Materials and Methods

### 2.1. Study Method

This study retrospectively evaluated patients admitted to the PICU, and a prospective approach was used to assess their current status. Thus, the study was designed as a cross-sectional retro-prospective analysis.

### 2.2. Study Population

Patients aged 2–18 years, diagnosed with asthma based on the Global Initiative for Asthma (GINA) criteria, without additional systemic diseases, and admitted to the PICU of Ankara Bilkent City Hospital between January 2013 and December 2022, were included in the study. This study involving human participants was conducted by the principles of the Declaration of Helsinki. Ethics approval was obtained from the Bilkent City Hospital Ethics Committee (Approval Number: TABED-1-24-118). Written informed consent was obtained from the caregivers of all patients prior to their participation in the study, and assent was also obtained for children over the age of 12. In our clinic, patients with a diagnosis of asthma, as indicated by International Classification of Diseases (ICD) codes, were evaluated for complaints such as coughing, shortness of breath, wheezing, chest pain, and exercise-induced dyspnea. The asthma diagnosis, based on ICD codes, was confirmed by a pediatric allergy and immunology specialist through physical examination and spirometry, by the GINA criteria [17].

### 2.3. Severe Asthma Attack Criteria Requiring PICU Monitoring (Age > 5 Years)

A severe asthma attack is characterized by orthopnea and agitation, with the patient only able to speak in single words. The respiratory rate exceeds 30 breaths per minute, and there is evident use of accessory respiratory muscles. The heart rate is above 120 beats per minute, and oxygen saturation (SpO_2_) is less than 90% on room air. Additional critical signs include a tendency to sleep, an altered level of consciousness, or the presence of a silent chest, all of which necessitate close monitoring and intensive care [2].

### 2.4. Severe, Life-Threatening Asthma Attack (Age < 5 Years)

A severe, life-threatening asthma attack in children under 5 years is characterized by agitation, confusion, or drowsiness. The respiratory rate exceeds 40 breaths per minute, and SpO_2_ is less than 92%. Heart rate is above 180 beats per minute in children aged 0–3 years and above 150 beats per minute in children aged 4–5 years. Additional critical signs include cyanosis and a silent chest, indicating the need for immediate intervention and intensive monitoring [2].

### 2.5. Study Data Collection and Follow-Up

Following discharge from the PICU, patients were invited for follow-up visits at the pediatric allergy and immunology outpatient clinic. Subsequently, their outpatient follow-up continued based on their asthma status and symptoms, with the frequency of clinic visits being planned according to each patient’s clinical condition. Asthma severity was classified according to medication requirements as mild (steps 1 and 2), moderate (steps 3 and 4), or severe (step 5), as recommended in the current GINA guidelines [2]. We defined asthma exacerbations as a worsening of symptoms. Exacerbations following hospitalization were characterized by increased symptoms such as shortness of breath, wheezing, coughing, and chest tightness, along with measurable declines in lung function. These exacerbations often necessitated changes in treatment, including increased use of short-acting bronchodilators, systemic corticosteroids, or, in severe cases, hospitalization [10].

The demographic and clinical characteristics of patients, intensive care support treatments, duration of hospital stays, atopic conditions, and nasal swab specimens were documented. The current clinical status of the patients was assessed by reviewing hospital records and conducting telephone interviews with their caregivers. A single follow-up phone interview was conducted, with patients being contacted an average of 3.5 years after discharge. However, patients who could not be reached or whose current asthma status was unknown were excluded from the study. The follow-up evaluation included aspects such as asthma emergency admissions within the past 12 months, ongoing asthma maintenance treatments, frequency of asthma exacerbations, and the level of asthma control based on the GINA guidelines [2].

### 2.6. Nasal Swab Collection and Analysis

The nasopharyngeal swab samples were taken for multiplex RT-PCR analysis within one hour following hospitalization; Respiratory Syncytial Virus (RSV), hRV, HIV, hPIV, hMPV, human bocavirus (HBV), coronavirus (CV), enterovirus (EV), and adenovirus (AV) PCR were studied. RNA extraction was performed with a Total Nucleic Acid Isolation Kit in Magnapure LC 2.0 isolation machine (Roche, Germany) or with EZ1 virus Mini Kit and Qiasymphony Virus/Bacteria Mini Kit in EZ1 Advanced XL and Qiasymphony isolation machine (Qiagen Gmbh, Hilden, Germany).

### 2.7. Statistic Analysis

Statistical analyses were conducted using SPSS software, version 25 (IBM Corporation, Armonk, NY, USA). Discrete variables were summarized as numbers and percentages, while continuous variables were presented as means with standard deviations (SDs) for normally distributed data and as medians with interquartile ranges (IQRs) for non-normally distributed data. Logistic regression analysis was employed to identify risk factors associated with stepping up asthma maintenance therapy. Variables with a *p*-value ≤ 0.20 in the univariate analysis were included in the multivariate analysis. Results were expressed as odds ratios (ORs) with 95% confidence intervals (CIs). A *p*-value < 0.05 was considered statistically significant for all analyses.

## 3. Results

### 3.1. Demographic and Clinical Characteristics

The study included 83 patients who met the inclusion criteria. The mean age of the patients was 6 years (±3.7), and the majority were male (*n* = 53, 63.9%). The average follow-up period after discharge was 3.4 ± 2.2 years. The mean duration of respiratory support in the PICU was 3.8 (±2.8) days, and the average duration of systemic steroid therapy was 4 (±1.5) days. The total length of hospital stays averaged 8.6 days (SD ± 4.2) (Table 1).

### 3.2. Recurrent ICU Admissions and Respiratory Support

Regarding the number of PICU admissions, 78.3% of the patients (*n* = 65) required only a single admission, 19.3% (*n* = 16) were admitted twice, and 2.4% (*n* = 2) required more than two admissions. During their PICU stay, 70.7% (*n* = 58) of the patients received intravenous magnesium sulfate and 74.4% (*n* = 61) received nebulized ipratropium bromide treatment. Among the patients included in the study, 8 (9.6%) required intubation, 2 (2.4%) received free-flow oxygen delivery, 20 (24.1%) were treated with a non-rebreathing mask, 42 (50.6%) required high-flow nasal cannula (HFNC) oxygen therapy, and 11 (13.3%) received non-invasive mechanical ventilation (NIMV) Table 2.

### 3.3. Respiratory Viral Panel Results

Pathogens were identified in 42 patients through respiratory viral panel testing. Rhinovirus was the most commonly isolated pathogen (*n* = 15), followed by co-infections of rhinovirus and respiratory syncytial virus (RSV) (*n* = 12, 8) (Table 3).

### 3.4. Post-Discharge Follow-Up

Of the 60 patients available for follow-up, 72.3% continued their care at pediatric allergy outpatient clinics. The follow-up data reflected the current evaluations, during which 60 patients were followed up in our clinic by pediatric allergy specialists. However, the remaining patients are being followed in other clinics by pediatric allergy immunologists, general pediatricians, or general practitioners, which accounts for the discrepancy in the numbers. Within the 12 months preceding follow-up, 67.5% (*n* = 56) of patients reported receiving current asthma treatment and 48.2% (*n* = 40) experienced an asthma exacerbation. Additional asthma symptoms experienced by the patients are detailed in Table 4.

### 3.5. Asthma Maintenance Therapy and GINA Steps

A comparison of asthma maintenance treatments pre-admission and post-discharge showed that 44.6% (n = 47) of the patients required an increase in their GINA treatment step after PICU admission, which was statistically significant (*p* < 0.001). No reduction in treatment steps was observed when patients were reassessed an average of 3.5 years after discharge (Table 5). A history of atopic dermatitis was identified as a significant risk factor for stepping up in GINA treatment steps in both univariate (*p* = 0.018) and multivariate (*p* = 0.03) analyses (Table 6).

### 3.6. Pulmonary Function Test Results

Pulmonary function test data were available for only 10 patients. No statistically significant differences were observed between pre-hospitalization and post-hospitalization pulmonary function test results. However, the overall proportion of completed pulmonary function tests relative to the total patient count was low (Table 7).

## 4. Discussion

This study, which investigated the course of asthma after admission to PICU, provided several important observations. Patients who experienced severe asthma exacerbations requiring PICU admission demonstrated a significant increase in their treatment steps according to GINA guidelines after discharge. Additionally, PICU admissions for asthma were identified as a significant risk factor for future severe exacerbations and hospitalizations. Respiratory viruses, particularly rhinovirus and RSV, were the most frequently isolated pathogens associated with exacerbations. Furthermore, a history of atopic dermatitis emerged as a notable predictor of increased asthma severity, as evidenced by step escalation in the GINA classification.

First of all, it was determined that patients experiencing asthma exacerbations requiring admission to PICU require a more advanced step of treatment according to GINA guidelines compared to initial management to maintain control of their asthma (*p* < 0.001). Following PICU admission, the maintenance treatments of 47 patients, accounting for 44.6% of these cases, were stepped up. In patients hospitalized with severe asthma exacerbations in the PICU, asthma maintenance therapy is reviewed at discharge, and the treatment step is typically escalated. However, in our study, when patients were reassessed an average of 3.5 years after discharge, it was determined that no reduction in the treatment step had been achieved. Based on this information, experiencing an asthma exacerbation requiring PICU was considered as a potential risk factor for poor asthma control. Indeed, numerous studies are showing that experiencing a severe asthma exacerbation is a significant predictor for future exacerbations [18,19,20]. In our study, we found that there was a need for more advanced treatment to maintain asthma control following a severe exacerbation. Children with poorly controlled asthma are at risk of exacerbations, school absence, hospital admission, and death, most of which are preventable [21].

In our study, male dominance was found to be evident at a rate of 63.9%, which is consistent with other studies [22,23,24,25]. PICU admissions for asthma constitute a significant proportion of all childhood asthma hospitalizations, ranging between 1% and 15%. Approximately 8% to 33% of this group require mechanical ventilation support. Apart from PICU admissions, childhood asthma accounts for a significant proportion of emergency department visits and other hospitalizations. Furthermore, research has emphasized that PICU admissions for asthma are a risk factor for both asthma-related hospitalizations and severe asthma exacerbations [7,26]. Abu Kishk et al. found that almost 25% of patients admitted to the PICU were hospitalized more than once and 17% were readmitted to the PICU [27]. Lee et al. evaluated 737 severe asthma exacerbations involving pediatric cases; 5% of them experienced two or more, and 2% experienced three or more exacerbations after the index event [28]. In our study, the rate of patients requiring readmission to the PICU due to asthma exacerbation following initial admission was found to be high, 21.7%. This finding is consistent with similar rates reported in other studies in the literature. In our study, we analyzed possible risk factors for repeated PICU admissions. However, no increased risk was identified. 

Severe RSV bronchiolitis has been linked to recurrent wheezing and asthma in later childhood, with evidence suggesting that RSV-induced changes in the airway epithelium, including the production of cytokines and chemokines, may contribute to the severity of subsequent asthma exacerbations [29,30,31].

As a result of the atopic march in allergic patients, the relationship between atopic dermatitis and asthma has been investigated in many aspects. These studies indicate that various factors such as genetic predisposition, immune system response, tissue-specific factors, and environmental triggers can influence this relationship. The immune system’s response, particularly the Th2 immune pathway, is implicated in both conditions. Dysregulation in this pathway can lead to allergic responses characteristic of both asthma and atopic dermatitis (AD). Tissue-specific factors, such as the local immune response and barrier dysfunction in the skin (in AD) or airways (in asthma), also contribute to the development and exacerbation of these conditions [32]. In our study, we found that a history of atopic dermatitis was identified as a risk factor for an increase in GINA severity steps, both in univariate and multivariate analyses. It is important to consider these results in the context of clinical management, as they highlight the need for increased monitoring and potentially more aggressive treatment strategies for patients with a history of AD. Further research may be warranted to explore the underlying mechanisms driving this association and to develop targeted interventions for this subgroup of asthma patients.

### Limitations

The limited measurement of objective data parameters such as spirometry to evaluate respiratory capacity during patient attacks and follow-ups can be considered a limitation of our study. The lack of data on patient compliance and inhaler technique in the article is one of our limitations. However, our strength lies in sharing single-center experiences regarding the long-term course of pediatric asthma patients with a history of PICU admission, for which limited data are available in the literature.

## 5. Conclusions

We found that admission to the PICU due to severe asthma exacerbation not only increases the risk of recurrent asthma exacerbations but also serves as a risk factor for stepping up maintenance treatment according to GINA guidelines during long-term follow-up.

## Figures and Tables

**Table 1:** Demographic features and asthma features.

Variables		
Age at the time of admission (year), mean ±SD		6 (±3.7)
Current age (year), mean ± SD		9.4 ± 4.5
Follow-up period from discharge (year), mean ± SD		3.4 ± 2.2
Birth weight, mean ± SD		2945 ± 542
Delivery method, *n*, (%)	Nsvy	44 (53)
	C/S	39 (47)
Male, *n*, (%)		53 (63.9)
Eosinophil count, median, (IQR)		200, (23–430)
Total IgE, median, (IQR)		50, (22–195)
Presence of atopy, *n*, (%)		17, (21.5)
Presence of hay fever, *n*, (%)		11, (13.9)
Presence of atopic dermatitis, *n*, (%)		14, (17.7)
Smoke exposure, *n*, (%)		14, (17.7)
Pet exposure, *n*, (%)		4, (4.8)
Atopic disease in the family, *n*, (%)		16, (20.5)
Pediatric allergy outpatient admission, *n*, (%)		60, (72.3)
Asthma exacerbation requiring PICU admission after PICU hospitalization, *n*, (%)		18, (21.7)

**Table 2:** Intensive care unit treatment details.

Intensive care effective treatment time (day), mean (SD)		3.8 ± 2.8	
Steroid treatment time (day), mean (SD)		4 ± 1.5	
Total duration of admission (day), mean (SD)		8.6 ± 4.2	
PICU Number of admissions, *n,* (%)		1	65, (78.3)
		2	16, (19.3)
		More than 2	2, (2.4)
Respiratory Support			
Intubation *n*, (%)			8, (9.6)
Free-flow oxygen delivery, *n*, (%)			2, (2.4)
Non-rebreathing mask, *n*, (%)			20, (24.1)
HFNC *, *n*,(%)			42, (50.6)
NIMV ^ϒ^, *n*, (%)			11, (13.3)
Medical Treatment			
Magnesium treatment, %			70.7
İpratropium bromide treatment, %			74.4

* HFNC: high-flow nasale cannulation, ^ϒ^ NIMV: non-invasive mechanic ventilation. PICU: pediatric intensive care unit.

**Table 3:** Isolation of respiratory viral panel.

	*N*, (%)
Rhinovirus	15, (19)
Respiratory Syncytial Virus (RSV)	12 (15.2)
Bocavirus (HBoV)	8, (10.1)
Adeno	1, (1.3)
nCoV2	2, (2.5)
SARS-CoV	2, (2.5)

**Table 4:** Asthma questionnaire.

	*n*, (%)
Wheezing in the last 12 months	38, (45.8)
Asthma exacerbations in the last 12 months	
1–3 exacerbations	33, (39.8)
4–12 exacerbations	7, (8.4)
Sleep disturbed due to wheezing?	
Once a week ≤	22, (26.5)
≥ More than once a week	3, (3.6)
Emergency clinic attendee due to asthma	36, (43.4)
Hospital admission due to asthma	11, (13.3)
Used steroids for asthma in the last 12 months?	13, (15.7)
Use of bronchodilators for asthma in the last 12 months?	55, (66.3)

**Table 5:** GINA step change.

		Before PICU Admission, *n*(%)	After PICU Admission, *n*(%)	*p*
GINA Step	1	56, (67.5)	41, (49.4)	<0.001
	2	12, (14.5)	-	
	3	14, (16.9)	33, (39.8)	
	4	1, (1.2)	9, (10.8)	

PICU: pediatric intensive care unit; GINA: the global initiative for asthma.

**Table 6:** GINA step increase risk factor.

	UNİVARİATE			MULTİVARİATE		
	OR	CI	*p*	OR	CI	*p*
Gender	0.65	0.26–1.61	0.36			
Presence of atopy	0.5	0.15–1.60	0.24			
Presence of allergic rhinitis	0.26	0.05–1.32	0.10	0.2	0.035–1.194	0.078
Atopic dermatitis	5.37	1.32–21.77	0.018	5.21	1.15–23.56	0.03
Smoke exposure	0.73	0.22–2.43	0.61			
Pet exposure	4.5	0.44–45.32	0.20	2.56	0.20–32.53	0.469
Famıly atopy hıstory	1.58	0.52–4.78	0.41			
Eosinophil count > 450 cells/μL	0.93	0.33–2.63	0.88			
Serum IgE levels > 150 IU/mL	1.01	0.33–3.03	0.97			

**Table 7:** Pulmonary function tests results.

	Before PICU Admission, Mean ± SD *N* = 10	After PICU Admission, Mean ± SD *N* = 10
FEV1,% predicted	87 ± 20	96 ± 10
FVC,% predicted	74 ± 19	76 ± 16
FEV1:FVC,%	94 ± 12	85 ± 17
FEF25–75,% predicted	93 ± 15	96 ± 22
PEF,% predicted	80 ± 11	75 ± 13

PICU: pediatric intensive care unit.

## Data Availability

The data presented in this study are available on request from the corresponding author.

## References

[1] National Asthma Education and Prevention Program, Third Expert Panel on the Diagnosis and Management of Asthma (2007). Section 2, Definition, pathophysiology and pathogenesis of asthma, and natural history of asthma. Expert Panel Report 3: Guidelines for the Diagnosis and Management of Asthma.

[2] Global Initiative for Asthma (2024). Global Strategy for Asthma Management and Prevention, 2024.

[3] Poddighe D., Brambilla I., Licari A., Marseglia G.L. (2018). Omalizumab in the therapy of pediatric asthma. Recent Pat. Inflamm. Allergy Drug Discov..

[4] Masoli M., Fabian D., Holt S., Beasley R., Global Initiative for Asthma (GINA) Program (2004). The global burden of asthma: Executive summary of the GINA Dissemination Committee report. Allergy.

[5] Ehteshami-Afshar S., FitzGerald J., Doyle-Waters M., Sadatsafavi M. (2016). The global economic burden of asthma and chronic obstructive pulmonary disease. Int. J. Tuberc. Lung Dis..

[6] Vos T., Flaxman A.D., Naghavi M., Lozano R., Michaud C., Ezzati M., Shibuya K., Salomon J.A., Abdalla S., Aboyans V. (2012). Years lived with disability (YLDs) for 1160 sequelae of 289 diseases and injuries 1990–2010: A systematic analysis for the Global Burden of Disease Study 2010. Lancet.

[7] Kaza V., Bandi V., Guntupalli K.K. (2007). Acute severe asthma: Recent advances. Curr. Opin. Pulm. Med..

[8] Mahesh S., Ramamurthy M.B. (2022). Management of acute asthma in children. Indian J. Pediatr..

[9] Joseph A., Ganatra H. (2024). Status Asthmaticus in the Pediatric ICU: A Comprehensive Review of Management and Challenges. Pediatr. Rep..

[10] O’Byrne P.M., Pedersen S., Lamm C.J., Tan W.C., Busse W.W. (2009). Severe exacerbations and decline in lung function in asthma. Am. J. Respir. Crit. Care Med..

[11] Mahase E. (2019). Asthma: Assess All Patients for Future Attack Risk and Tailor Care Accordingly, New Guideline Advises.

[12] Grunwell J.R., Travers C., Fitzpatrick A.M. (2018). Inflammatory and comorbid features of children admitted to a PICU for status asthmaticus. Pediatr. Crit. Care Med..

[13] Van Den Bosch G.E., Merkus P.J., Buysse C.M., Boehmer A.L., Vaessen-Verberne A.A., Van Veen L.N., Hop W.C., De Hoog M. (2012). Risk factors for pediatric intensive care admission in children with acute asthma. Respir. Care.

[14] Papi A., Blasi F., Canonica G.W., Morandi L., Richeldi L., Rossi A. (2020). Treatment strategies for asthma: Reshaping the concept of asthma management. Allergy Asthma Clin. Immunol..

[15] Engelkes M., Janssens H.M., de Jongste J.C., Sturkenboom M.C., Verhamme K.M. (2015). Medication adherence and the risk of severe asthma exacerbations: A systematic review. Eur. Respir. J..

[16] Louie S., Morrissey B.M., Kenyon N.J., Albertson T.E., Avdalovic M.V. (2012). The critically ill asthmatic: From ICU to discharge. Bronchial Asthma: A Guide for Practical Understanding and Treatment.

[17] Education N.A., Program P. (2007). Expert panel report 3 (EPR-3): Guidelines for the diagnosis and management of asthma-summary report 2007. J. Allergy Clin. Immunol..

[18] Haselkorn T., Zeiger R.S., Chipps B.E., Mink D.R., Szefler S.J., Simons F.E.R., Massanari M., Fish J.E. (2009). Recent asthma exacerbations predict future exacerbations in children with severe or difficult-to-treat asthma. J. Allergy Clin. Immunol..

[19] Miller M.K., Lee J.H., Miller D.P., Wenzel S.E., Group T.S. (2007). Recent asthma exacerbations: A key predictor of future exacerbations. Respir. Med..

[20] Chipps B.E., Zeiger R.S., Borish L., Wenzel S.E., Yegin A., Hayden M.L., Miller D.P., Bleecker E.R., Simons F.E.R., Szefler S.J. (2012). Key findings and clinical implications from The Epidemiology and Natural History of Asthma: Outcomes and Treatment Regimens (TENOR) study. J. Allergy Clin. Immunol..

[21] Levy M.L., Andrews R., Buckingham R., Evans H., Francis C., Houston R., Lowe D., Nasser S., Paton J., Puri N. (2014). Why Asthma Still Kills: The National Review of Asthma Deaths (NRAD).

[22] Bisgaard H., Szefler S. (2007). Prevalence of asthma-like symptoms in young children. Pediatr. Pulmonol..

[23] Kuehni C.E., Strippoli M.-P.F., Low N., Brooke A.M., Silverman M. (2007). Wheeze and asthma prevalence and related health-service use in white and south Asian pre-school children in the UK. Clin. Exp. Allergy..

[24] Martinez F.D., Wright A.L., Taussig L.M., Holberg C.J., Halonen M., Morgan W.J., Associates G.H.M. (1995). Asthma and wheezing in the first six years of life. N. Engl. J. Med..

[25] Mukherjee M., Cunningham S., Bhuia M.R., Lo T.-Y.M., Been J.V., Sheikh A. (2022). Asthma in paediatric intensive care in England residents: Observational study. Sci. Rep..

[26] Alvarez G., Schulzer M., Jung D., Fitzgerald J. (2005). A systematic review of risk factors associated with near-fatal and fatal asthma. Can. Respir. J..

[27] Abu-Kishk I., Polakow-Farkash S., Elizur A. (2016). Long-Term Outcome After Pediatric Intensive Care Unit Asthma Admissions.

[28] Lee T.Y., Petkau J., Sadatsafavi M. (2022). Long-term natural history of severe asthma exacerbations and their impact on the disease course. Ann. Am. Thorac. Soc..

[29] Driscoll A.J., Arshad S.H., Bont L., Brunwasser S.M., Cherian T., Englund J.A., Fell D.B., Hammitt L.L., Hartert T.V., Innis B.L. (2020). Does respiratory syncytial virus lower respiratory illness in early life cause recurrent wheeze of early childhood and asthma? Critical review of the evidence and guidance for future studies from a World Health Organization-sponsored meeting. Vaccine.

[30] Piedimonte G., Perez M.K. (2008). Role of early-life environmental influences in the development of asthma. How painful is it when you catch a bad cold too early?. J. Asthma.

[31] Manti S., Piedimonte G. (2022). An overview on the RSV-mediated mechanisms in the onset of non-allergic asthma. Front. Pediatr..

[32] Paller A.S., Spergel J.M., Mina-Osorio P., Irvine A.D. (2019). The atopic march and atopic multimorbidity: Many trajectories, many pathways. J. Allergy Clin. Immunol..

